# Silk Fibroin-Coated Liposomes as Biomimetic Nanocarrier for Long-Term Release Delivery System in Cancer Therapy

**DOI:** 10.3390/molecules26164936

**Published:** 2021-08-15

**Authors:** Chanon Suyamud, Chanita Phetdee, Thanapak Jaimalai, Panchika Prangkio

**Affiliations:** 1 Master’s Degree Program in Chemistry, Faculty of Science, Chiang Mai University, Chiang Mai 50200, Thailand; chanon_suy@cmu.ac.th; 2 Department of Chemistry, Faculty of Science, Chiang Mai University, Chiang Mai 50200, Thailand; chanita_phet@cmu.ac.th (C.P.); thanapak.nj@gmail.com (T.J.); 3 Doctor of Philosophy Program in Chemistry, Faculty of Science, Chiang Mai University, Chiang Mai 50200, Thailand; 4 Center of Excellence in Materials Science and Technology, Faculty of Science, Chiang Mai University, Chiang Mai 50200, Thailand

**Keywords:** silk fibroin, liposome coating, liposomes, biomimetic nanocarrier, drug delivery, doxorubicin, breast cancer

## Abstract

Despite much progress in cancer therapy, conventional chemotherapy can cause poor biodistribution and adverse side-effects on healthy cells. Currently, various strategies are being developed for an effective chemotherapy delivery system. Silk fibroin (SF) is a natural protein used in a wide range of biomedical applications including cancer therapy due to its biocompatibility, biodegradability, and unique mechanical properties. In this study, SF-coated liposomes (SF-LPs) were prepared as a biomimetic drug carrier. Physicochemical properties of SF-LPs were characterized by Fourier-transform infrared spectroscopy (FTIR), dynamic light scattering, zeta potential measurement, and transmission electron microscopy (TEM). In vitro release of SF-LPs loaded with doxorubicin (DOX-SF-LPs) was evaluated over 21 days. Anticancer activity of DOX-SF-LPs was determined against MCF-7 and MDA-MB231 cells using the MTT assay. SF-LPs containing 1% SF exhibited favorable characteristics as a drug carrier. SF coating modified the kinetics of drug release and reduced the cytotoxic effect against L929 fibroblasts as compared to the uncoated liposomes containing cationic lipid. DOX-SF-LPs showed anticancer activity against breast cancer cells after 48 h or 72 h at 20 μM of DOX. This approach provides a potential platform of long-term release that combines biocompatible SF and phospholipids for cancer therapy, achieving efficient drug delivery and reducing side-effects.

## 1. Introduction

Among various types of cancer, breast cancer is the most common cancer affecting women and is still one of the leading causes of cancer death in 2021 [1]. Despite much progress and advancement in cancer treatment, conventional chemotherapy still encounters problems due to a lack of specificity of the drug and cytotoxicity, resulting in side-effects and drug resistance in tumor cells [2]. To diminish the adverse effects of chemotherapeutic drugs, various strategies have been investigated to improve bioavailability and specificity of drug action, thereby reducing the harmful side-effects of the drugs, especially for long-term administration [3]. In general, chemotherapeutic drugs at therapeutic concentrations are required to remain at the target site for a long period of time without causing cytotoxic effects on other cells [4]. A number of drug delivery systems, including polymeric nanoparticles, liposomes, micelles, and hydrogels, have been employed for sustained drug release systems [5]. Liposomes are considered the most common nanocarriers used in drug delivery due to the similarity of the lipid bilayer to the cell membranes. Liposomes also offer several advantages including biocompatibility, self-assembly, ability to encapsulate a wide range of drugs or macromolecules, and possibility of surface modification with polymers or ligands which govern physicochemical and biophysical properties of liposomes. Despite their versatility, some reports have shown that liposomes may trigger immune responses, depending on the characteristics of the liposomes, and they tend to be rapidly eliminated by the reticuloendothelial system (RES), thus limiting their therapeutic efficacy [6,7].

To improve functionality of liposomes, the liposomal surface can be modified by conjugation with various moieties to extend blood circulation time and decrease the adsorption of blood proteins. In particular, liposomes can be coated with a biological matrix such as alginate, chitosan, pectin, collagen, or fibroin [8,9,10,11,12,13,14] to mimic the structure of biological cells due to the biocompatibility, biodegradability, and bioavailability of these biomaterials [15]. Moreover, these polymeric materials provide steric hindrance to prevent the aggregation of liposomes and inhibit the absorption of plasma proteins and RES uptake.

**Figure 1:**
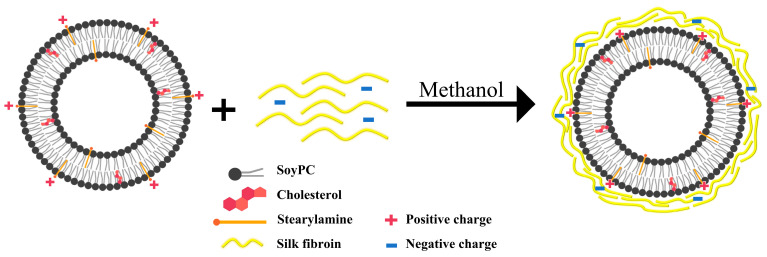
Schematic representation of liposomes containing soy phosphatidylcholine (soy PC), cholesterol (CH), and cationic stearylamine (SA) coated with SF via electrostatic interaction.

In the past few decades, silk proteins have attracted great attention as promising natural polymers for biomedical and pharmaceutical applications [16,17,18]. Silk fibroin (SF) is a natural fiber protein produced by *Bombyx mori* mulberry silkworm that has been extensively used in biomedical applications due to its biocompatibility, nontoxicity, non-immunogenicity, and the ability to adhere to the cell membrane [19,20]. In this study, SF was used for coating liposome surface to prolong circulation time and improve drug efficacy over a long period. Owing to the advantages of both liposomes and SF as biocompatible materials, SF-coated liposomes (SF-LPs) loaded with an anticancer drug, doxorubicin (DOX), were developed as a drug carrier. Additional layers of SF could provide a physical barrier to prevent burst release of cargo from the liposomes in physiological environments. Despite a few studies using a similar approach [3,19,21], this work underlies the preparation method of SF-LPs loaded with DOX targeting breast cancer cells, providing a smaller diameter range (<150 nm), which has been reported to remain in the tumor microenvironment for an extended time [22]. Thus, this biomimetic approach demonstrates a hybrid platform with the possibility of increasing bioavailability for long-term drug release used in cancer therapy. As shown in Figure 1, the liposome was mainly composed of soy phosphatidylcholine (PC), cholesterol (CH), and positively charged stearylamine (SA), which is a long-chain alkyl amine providing an electrostatic interaction with the negative charges on SF. Additionally, DOX-loaded liposomes (DOX-LPs) were prepared via the transmembrane ammonium sulfate gradient approach [23], followed by SF coating with 0.5–2% SF, resulting in DOX-loaded SF-LPs (DOX-SF-LPs). For optimization of the sustained-release drug delivery system, the encapsulation efficiency (%EE), cytotoxicity, and DOX release profile were investigated. Our study demonstrates that DOX-SF-LPs exhibited anticancer activity against breast cancer cell lines, MCF-7 and MDA-MB231, especially after 48 h. Although the anticancer effect of DOX-SF-LPs was not prominent as compared to that of free DOX or DOX-LPs, SF coating markedly reduced cytotoxicity in normal cells and exhibited long-term drug release, offering an alternative strategy for cancer therapy.

## 2. Results and Discussion

### 2.1. Characterization of SF Extracts

**Figure 2:**
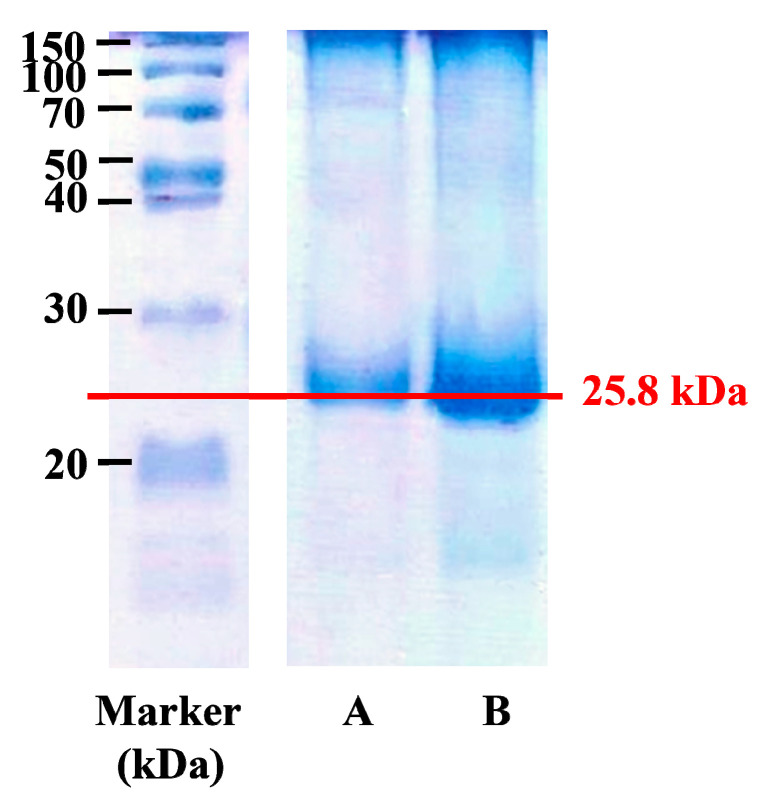
SDS-PAGE analysis of SF extracts at (**A**) 1 mg/mL and (**B**) 3 mg/mL, as determined by the measurement of absorbance at 280 nm. The estimated molecular weight of SF was 25.8 kDa.

*Bombyx mori* silk cocoons consist of two major proteins, fibroin and sericin. In the process of SF extraction, SF was separated from silk cocoons by degumming to remove sericin. The neutral salt LiBr (9.3 M) was required to completely dissolve silk fibers for 4 h by disrupting peptide bonds in the SF molecular chains. Furthermore, the temperature in the reaction should not exceed 70 °C to prevent protein denaturation and gelation that may occur after dialysis. The concentration of the extracted SF was approximately 10.54% ± 2.93% *w/v*. As confirmed by SDS-PAGE (see Figure 2), the major band of extracted SF was observed at approximately 25.8 kDa, which represents the light chain of SF [24], suggesting that the light-chained SF fraction was not completely degraded.

**Figure 3:**
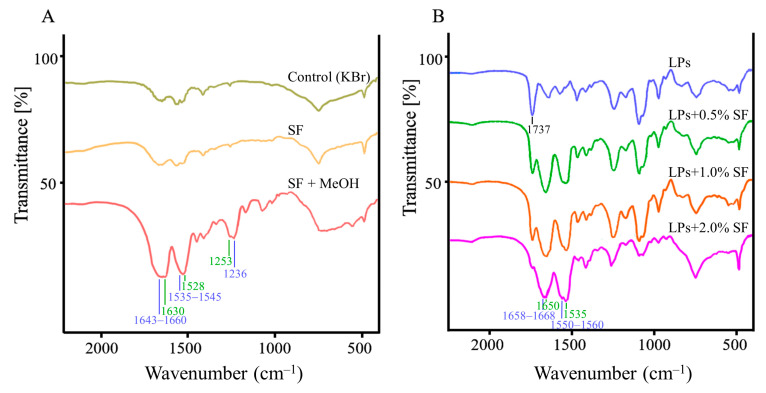
FTIR spectra of SF and SF-LPs. (**A**) SF with and without treatment of methanol; (**B**) uncoated liposomes and SF-LPs using SF of 0.5–2.0%.

Fourier-transform infrared (FTIR) spectroscopy was used to characterize the secondary structure of SF before and after coating on liposomes. As a result, the secondary structure of SF was clearly observed after treatment with methanol, as shown in Figure 3A. FTIR spectra of SF treated with methanol demonstrated sharp peaks of β-sheet conformation at 1630 cm^−1^, 1520 cm^−1^, and 1265 cm^−1^, while the minor bands of α-helix and random coil conformations were shown at 1650–1670 cm^−1^, 1535–1545 cm^−1^, and 1230 cm^−1^. The predominant β-sheet structure of SF was induced by 50% methanol via disruption of hydrophobic hydration of regenerated SF, resulting in self-assembly of the less-ordered structure into a β-sheet structure [25,26]. Upon coating liposomes with SF, similar absorption FTIR spectra of SF-LPs were observed at 1630 cm^−1^, 1520 cm^−1^, and 1265 cm^−1^, indicating the presence of SF with a β-sheet conformation. In addition, Figure 3B demonstrates the band of 1740 cm^−1^ for the ester group of the lipid composition (C=O stretching) for all liposomes samples.

### 2.2. Characterization of Liposomes Modified with SA

**Figure 4:**
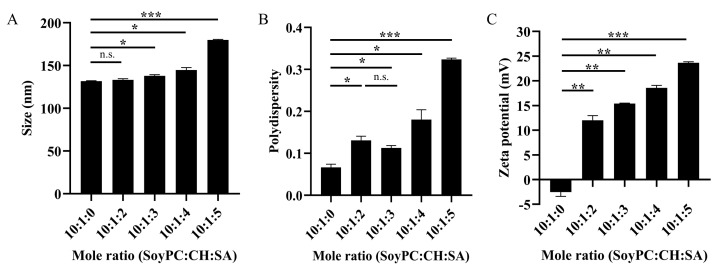
Physicochemical properties of liposomes with various amount of stearylamine (SA) in the liposomes containing soyPC, CH, and SA, including (**A**) particle size, (**B**) polydispersity index, and (**C**) zeta potential. Data are presented as the mean ± SD (*n* = 3). n.s. denotes not significant; * *p* < 0.05; ** *p* < 0.01; *** *p* < 0.001.

To obtain biomimetic liposomes, biological polymer SF was used to modify the liposome surface to provide the release of the cargo at a controlled rate [27]. Since the overall charge of SF was slightly negative, the positively charged surface of liposomes could provide the electrostatic interactions and facilitate SF coating. In this work, the liposomes comprising soy PC and CH had a neutral charge at a pH of approximately 7. Cationic alkyl amine SA was added to the mixture to provide positive charges on the LP surfaces. To determine the optimal conditions of liposomes before SF coating, the physicochemical properties of lipid compositions at various mole ratios were characterized. Figure 4 demonstrates the influence of SA on size, polydispersity, and surface charges of liposomes, which are important factors for evaluating the stability of drug carriers [22]. In this study, the sizes of all liposomes were in the diameter range of 120–180 nm, which are considered suitable for passively targeting tumor tissues via an enhanced permeability and retention effect [22]. The diameter of the liposome increased upon increasing the concentration of SA, as shown in Figure 4A. In liposomal drug delivery, a polydispersity index (PDI) of 0.3 or below is acceptable for long-term circulating nanocarriers, such as in tumor targeting [22]. As shown in Figure 4B, the PDI values were below 0.3 for most conditions, except for that of soy PC:CH:SA (10:1:5). In the absence of SA, the liposomes contained a small proportion of negative charges attributed to zwitterionic soy PC. The zeta potentials of liposomes consisting of SA were positive values (+10 to +25 mV) and significantly increased as the positively charged SA concentration increased (see Figure 4C). Importantly, the zeta potential of liposomes in this condition was ~+15 mV, which was significantly higher than that of liposomes without modification by SA, and this value was sufficient for providing liposomal stability and preventing aggregation [28]. Although the mole ratios of soy PC:CH:SA at 10:1:4 and 10:1:5 offered the high values of zeta potential, these conditions were not practical because of the high density of lipid, which resulted in difficulty in the extrusion of liposomes during preparation. These conditions with a high amount of SA might not be appropriate for SF-LP preparation. On the basis of the results altogether, soy PC:CH:SA at 10:1:3 was chosen as the main composition of the liposomes modified with SA before SF coating.

### 2.3. Physicochemical Properties of SF-LPs

After achieving the optimal ratio of liposomes, the liposomes of soy PC:CH:SA (10:1:3) were coated with the extracted SF at 0.5%, 1%, and 2%. As shown in Table 1, the uncoated liposomes in the absence of SF were found to exhibit ~136 nm in diameter, +16.4 mV of zeta potential, and PDI values of 0.135–0.238. As the SF concentration increased, the sizes of SF-LPs increased, while the zeta potentials decreased, indicating that multiple layers of SF were being coated on the liposome surfaces. Our findings demonstrate that SF-LPs exhibited lower zeta potential values than the uncoated liposomes since the SF reduced the positive charges of SA on the liposome surface upon binding. During the coating process, methanol was added to liposomes to cause SF denaturation and membrane disruption, allowing SF to adhere tightly on the liposome surface via hydrophobic interactions [21,29]. Without the addition of methanol, the SF-LPs became larger in size (>200 nm) and exhibited higher PDI values (>0.5) (see Supplementary Materials, Table S1), suggesting that methanol is a key factor for achieving homogenous SF-LPs with uniform surfaces. In this study, SF coating provided the SF-LPs with a significantly larger diameter and sufficient zeta potential for maintaining stability of liposomes as a lipid-based colloidal delivery system [28]. Nevertheless, the size of the SF-LPs with 2.0% SF exceeded 150 nm, which may not be efficiently internalized into tumor cells via enhanced permeability and retention [22]. Thus, 1% *w*/*v* SF was considered suitable for coating SF-LPs with enhanced physicochemical properties and stability.

### 2.4. Morphology of SF-LPs

**Figure 5:**
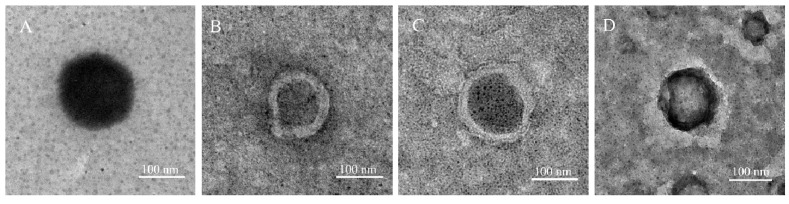
Representative TEM images of liposomes and SF-LPs with different SF concentrations. (**A**) Uncoated liposomes and SF-LPs when using (**B**) SF 0.5%, (**C**) SF 1.0%, and (**D**) SF 2.0%. In the presence of SF, a multilayer appearance was observed on the surface of liposomes.

To confirm the physical characteristics of SF-LPs, transmission electron microscopy (TEM) imaging of all samples was performed. TEM images revealed the morphology of spherical particles of uncoated liposomes and core–shell SF-LPs. Multilayered characteristics were observed only in SF-LPs, as shown in Figure 5. Moreover, the spectrophotometric determination (A_280_) was used to confirm the presence of SF in the coated SF-LPs. The dark appearance in the cores of liposomes was likely due to ionic interaction of phosphotungstic acid as a negative staining agent and the positive charges on the liposomes [30]. The sizes of liposomes obtained from the TEM results were comparable to those obtained from DLS studies, as summarized in Table 1. According to the TEM images, the SF-LPs (1% SF) containing SA clearly demonstrated a relatively smooth surface when compared to that without SA (see Supplementary Materials, Figure S1), indicating the importance of cationic SA in SF coating. However, the other physicochemical properties need to be taken into consideration for in vivo drug delivery systems.

### 2.5. In Vitro Cytotoxicity

**Figure 6:**
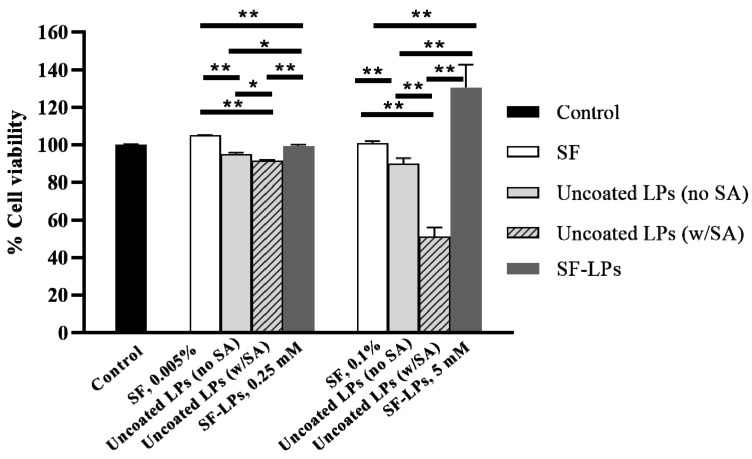
Cell viability of L929 fibroblasts when treated with components of uncoated liposomes (LPs) and SF-LPs. The uncoated liposomes containing SA showed a reduction in cell viability by 20%, whereas SF-LPs could enhance cell proliferation. The lipid compositions were soy PC, CH, and SA at 10:1:3 (molar ratio), with total phospholipid concentrations of 0.25 mM and 5 mM; SF 1% (*w/v*) was used for coating liposomes. ** *p* < 0.01; * *p* < 0.05.

Biomimetic drug carriers are developed to promote drug efficacy, cellular uptake, and sustainability of drug release to target cells. Biological polymers used as coating materials on liposome surfaces should also be biocompatible and biodegradable, as well as exhibit a low cytotoxic effect on normal cells. To investigate potential cytotoxicity, the liposome samples with and without SF coating were examined on L929 fibroblasts using the MTT assay. In this study, soy PC and CH were used as the main components of liposomes, whereas a cationic lipid, SA, was incorporated into the liposomes to provide an electrostatic interaction with SF as a coating material. Without SF coating, the liposomes significantly reduced the cell viability, especially for that containing SA, in a dose-dependent manner as shown in Figure 6. The uncoated liposomes exhibited cytotoxicity, which was mainly attributed to the SA component. Cationic liposomes are generally utilized for enhancing the cellular uptake via electrostatic interactions with the cell membrane of tumor cells, which express a high proportion of negative charges [31]. Although SA is a cationic agent commonly used in drug and gene delivery systems, toxicity and hemolytic activity of SA at critical density have been reported [32,33]. To improve the biocompatibility of cationic liposomes, conjugation of lipids with nontoxic molecules, such as amino acids or polypeptides, is a promising approach. Here, we demonstrate that SF coating provided a reduction in the positive charges of SF-LPs (see Table 1). In comparison with the uncoated liposomes, the cell viability of SF-LPs was improved significantly, while SF itself exhibited no cytotoxic effect on the fibroblasts. As previously reported, SF-based materials are known to promote cell adhesion and cell proliferation of fibroblasts [3,34]. Nevertheless, the cytotoxicity of liposomes containing SA could be observed when the concentration of total lipid was in micromolar range. Thus, the SF-LPs offer advantages of lowering the toxicity of the drug carrier in the delivery system, but they should be used within the threshold limit of lipid concentrations.

### 2.6. Encapsulation of Anticancer Drug in SF-LPs

Liposomal drug delivery systems have been extensively investigated for cancer therapy. An ideal delivery system should provide stability of the drug cargo with sustained drug release and targeting specificity to minimize adverse effects of the drug [17]. For encapsulation of chemotherapeutic DOX, SF-LPs were utilized to form the core–shell structure that provides sustained release. DOX was loaded into the SF-LPs via the transmembrane pH gradient method, which is a common approach for drug encapsulation through an ion gradient to achieve a high %EE [35]. The gradient of an ionized and a nonionized salt is created between the internal and external compartments of liposomes via the exchange process until equilibrium is reached [6]. In this study, without SF coating, the obtained %EE of DOX was approximately 96%, suggesting that DOX was efficiently loaded into the uncoated liposomes. Interestingly, when DOX was loaded into the SF-LPs, using 0.5–2.0% SF (*w*/*v*), the %EE of DOX in SF-LPs slightly decreased to 88–95% (see Table 1), which was still considered high compared to drug-loaded liposomes using the simple passive loading method [5]. Moreover, the %EE in the SF-LPs slightly decreased as the % SF used for coating increased, possibly because the SF coating outside may adsorb the drug molecules, thus reducing the amount of loaded cargo in the liposomes.

### 2.7. In Vitro Release of DOX

**Figure 7:**
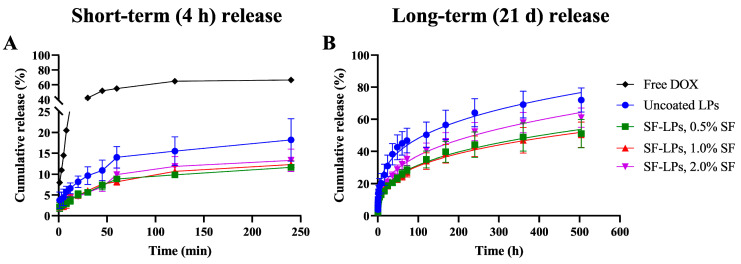
Comparison of in vitro release of DOX from uncoated liposomes and SF-LPs in PBS, pH 7.4 at 37 °C, over two periods. (**A**) Short-term release over the first 4 h (240 min) of drug release. (**B**) Long-term release over 21 days (500 h). The long-term release profiles were fitted with the Korsmeyer–Peppas kinetic model, M_t_/M_∞_ = K_kp_ × *t^n^*, where M_∞_ is the amount of drug at the equilibrium state, M_t_ is the amount of drug released over time *t*, K_kp_ is the constant incorporating structural and geometrical characteristics of the nanocarrier, and *n* is the diffusional exponent or drug release that determines drug release mechanism. The fitting curves demonstrate that the release behavior of DOX from uncoated liposomes and SF-LPs were governed by Fickian diffusion mechanism as a matrix system.

The in vitro release of DOX that was encapsulated in different formulations of SF-LPs was evaluated using a dialysis bag under physiological conditions (PBS, pH 7.4). DOX release profiles were compared between short-term (4 h) and long-term (21 days) periods, as shown in Figure 7. In the first 4 h, the % release of DOX was clearly distinct between the uncoated and coated SF-LPs, suggesting that the SF coating provides a barrier for DOX to be released from the SF-LPs. In contrast, the release of free DOX in the absence of liposomes, representing conventional drug release, displayed an initial burst release of nearly 70% at 4 h, but the release of free DOX could not be determined after 8 h because the precipitation of released DOX in PBS (pH 7.4) was observed due to its poor solubility in PBS. Considering the long-term release over a period of 21 days, when most chemotherapeutic drugs are required to be retained in the circulation system [4], the release profile of DOX from the uncoated liposomes reached a maximum of 70%, suggesting that the SF-LPs may affect the retardation of DOX release. Among all conditions of SF-LPs, 0.5% and 1.0% SF coating provided similar release rates, whereas a higher rate was observed in 2.0% SF, possibly due to the aggregation of SF-LPs. According to the results from zeta potential analysis and TEM imaging, a high SF concentration may lead to the instability of liposomes, thus losing drug retention capacity.

To gain an understanding of the drug release mechanism of SF-LPs, mathematical models were used to describe the behavior of the encapsulated drug from the delivery systems. The drug release process depends on several factors such as drug solubility, drug stability, physicochemical properties of the drug carriers, release environment, and possible interactions among these factors [36,37]. In this study, the experimental data of long-term (21 days) release showed a good fit (*R^2^* = 0.99) to the Korsmeyer–Peppas kinetic model, which is a power law model for studying drug release from polymeric systems [38]. The results of fitting parameters are summarized in Table 2. According to the fitting results, the extrapolated release exponent (*n*) values ranged from 0.2877 to 0.3287 for all formulations (less than 0.43), suggesting a Fickian diffusion mechanism of drug from a spherical matrix [39]. The rate constant for DOX diffusion from the uncoated liposomes (K_kp_ = 12.80) was higher than that of the coated SF-LPs (K_kp_ = 6.953–7.993). Although SF-LPs using SF at 0.5% to 2% may not have shown a distinct release behavior, it was clearly seen that the polymeric SF used for coating on the liposome surface affected drug release. The SF matrix could stabilize the drug and provide extra layers to slow down drug release, especially at the early stage. Based on the biomimetic approach, SF could prevent the interaction between nanocarriers and plasma proteins in blood circulation. Moreover, SF-LPs could enhance the cell adhesion and uptake behaviors because of the fibrous structure and unique mechanical properties of the SF [19,21]. Therefore, SF is a promising biological polymer that can be useful for coating material, regulating the cell adhesion, and enabling long-term drug release from a nanocarrier. 

### 2.8. Anticancer Activity of DOX in SF-LPs

**Figure 8:**
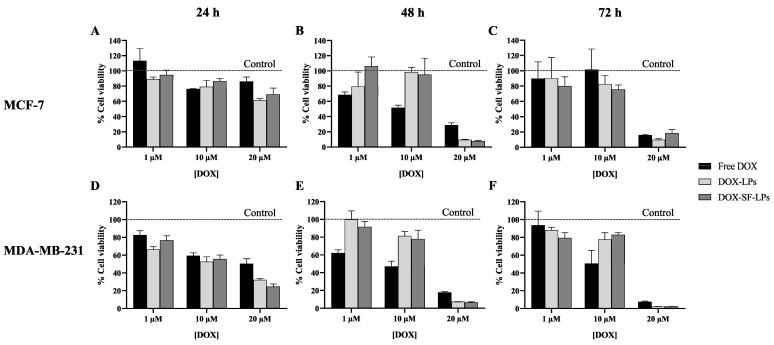
Cell viability of (**A**–**C**) MCF-7 and (**D**–**F**) MDA-MB-231 when exposed to DOX as a free drug and encapsulated in liposomes (DOX-LPs) or SF-coated liposomes (DOX-SF-LPs). SF-LPs were composed of soy PC:CH:SA (10:1:3) and 1% SF. The cells were treated with DOX in all forms at the final concentrations of 1, 10, and 20 μM for 24 h, 48 h, and 72 h. The percentage of cell viability was determined using the MTT assay. The cell viability was normalized within the control (PBS) of each incubation period as indicated by dashed lines (100% cell viability). Data are presented as the mean ± SEM, *n* = 3.

As a chemotherapeutic drug, DOX can inhibit cell proliferation by causing DNA damage and generating reactive oxygen species which can trigger oxidative stress and apoptosis. The generated free radicals can also attack the components of cell membranes, resulting in cell death [40]. The breast cancer cell lines, MCF-7 and MDA-MB-231, were exposed to the uncoated DOX-LPs, DOX-SF-LPs, and free DOX at 1 μM, 10 μM, and 20 μM of DOX for 24 h, 48 h, and 72 h using culture medium as a negative control. Figure 8 illustrates the cell viability of cancer cells upon treatment of DOX as determined by the MTT assay. The cell viability of both cancer cell lines slightly decreased by 20–40% after 24 h treatment of 10–20 μM DOX, while MDA-MB231 cells showed more sensitivity to DOX than MCF-7. Although the anticancer effect of DOX was not clearly observed at 24 h for DOX in both free drug and liposomal forms, DOX at 20 μM in SF-LPs exhibited higher anticancer activity than free DOX at 48 h and 72 h. The cytotoxicity of DOX was less evident when using DOX at the lower concentrations (1 or 10 μM), especially at 72 h where the cell proliferation increased after a long period. Moreover, free DOX had a shorter half-life than the drug entrapped in liposomes [41], resulting in a lowered therapeutic effect at 72 h. Because of its adhesive properties, SF coating could enhance the cell adhesion and stabilize the drug within the SF-LP matrix, thus slowing down drug release. This study demonstrates that DOX-SF-LPs significantly increased anticancer activity at 20 μM DOX after a longer period of exposure. Notably, a high concentration of DOX is required for encapsulation in SF-LPs to exhibit in vitro anticancer activity. 

Previously, Cheema et al. reported a similar approach of using SF-LPs to encapsulate anticancer agent emodin and showed an increase in cellular uptake and efficacy in killing breast cancer cells after 96 h exposure as compared to the uncoated liposomes [19]. In our study, DOX which is a relatively hydrophilic compound, tended to diffuse from the SF-LPs more rapidly than emodin. Hence, there was no significant difference in DOX release kinetics and anticancer activity between DOX-LPs and DOX-SF-LPs. The interactions of SF coatings around the SF-LPs may slightly modify DOX release in SF-LPs but clearly did not interfere with the drug action. Furthermore, SF-LPs exhibited less toxicity in the normal cells than the uncoated liposomes. Owing to its biocompatibility and unique mechanical properties, SF can be used as a coating material on liposome surfaces to provide a long-term delivery system with reduced toxicity to normal cells. The SF coating can potentially be used in combination with different drug carriers to enhance the drug release control [18]. Nevertheless, the physicochemical properties of the drug and nanocarriers need to be further investigated to achieve overall benefits in efficiency of the drug delivery system. 

## 3. Materials and Methods

### 3.1. Materials

Cocoon shells of silkworms were obtained from the Queen Sirikit Department of Sericulture Center, Ministry of Agricultural and Cooperatives (Chiang Mai, Thailand. Soy PC extract, 95% was purchased from Avanti Polar Lipids, Inc (Alabaster, AL, USA). All reagents and solvents were purchased from Sigma-Aldrich (St. Louis, MO, USA) unless otherwise stated.

### 3.2. Extraction of SF

Silk cocoons were cut into small pieces and boiled in 0.02 M Na_2_CO_3_ for 30 min to remove water-soluble sericin. After degumming, the silk fibers were rinsed with deionized water three times and dried overnight. Then, the silk fibers were dissolved in a 9.3 M LiBr solution for 4 h at 60 °C. The SF solution was then dialyzed by dialysis cassette, 20 kDa MWCO (Thermo Fischer Scientific, Waltham, MA, USA), for 3 days to remove salt [42] and centrifuged at 10,000 rpm for 20 min to remove insoluble fibrous debris. The dialyzed SF solution was collected and stored at 4 °C until used.

### 3.3. Characterization of SF Extracts

#### 3.3.1. Determination of Protein Concentration and Molecular Weight

After SF extraction was accomplished, the concentration of SF was determined by measuring absorbance at 280 nm using a Thermo Scientific™ Evolution 201/220 UV/Vis Spectrophotometer (Thermo Fisher Scientific, Waltham, MA, USA). Bovine serum albumin was used as a protein standard [43]. The molecular weight of SF was determined by SDS-PAGE using 15% gel. Electrophoresis was performed by using a constant voltage (100 V) at room temperature in 1× solution of Tris–glycine running buffer containing 1% SDS for approximately 90 min. After SDS-PAGE, the gel was stained with Coomassie Brilliant Blue R-250 and destained with acetic acid solution.

#### 3.3.2. Determination of Secondary Structure of SF 

The secondary structure of SF was analyzed by FTIR spectroscopy. The lyophilized samples, including SF, uncoated liposomes, and SF-LPs, were mixed with KBr powder and pressed into a solid pellet using a manual hydraulic press. Then, the solid pellets were measured under transmission mode using an FTIR spectrophotometer (Bruker-Tensor27, Billerica, MA, USA). FTIR spectra were recorded in the spectral range of 500–2000 cm^−1^ representing the secondary structures of α-helix, β-sheet, and random coil of the SF protein.

### 3.4. Preparation of Liposomes and SF-LPs

Liposomes were prepared using the thin-film hydration method. A mixture of lipids containing soy PC, CH, and SA at various mole ratios was dissolved in chloroform and dried under vacuum using a rotary evaporator to obtain a thin film of lipid. The lipid film was hydrated with PBS pH 7.4 and incubated for 2 h at 37 °C. The liposomes were subjected to 21 extrusions through a 0.1 µm polycarbonate membrane. Subsequently, the SF solution at three concentrations (0.5%, 1.0%, and 2.0%) was added to the liposome solution and gently stirred. The mixed solution was incubated at 4 °C overnight (approximately 12 h). Then, 50% methanol was added to the solution in a 1:5 ratio (methanol–liposome) and incubated for 10 min. Subsequently, methanol was removed by two centrifugations at 1000× *g* for 30 min using Amicon^®^ ultra centrifugal filters, 100 kDa MWCO (Merck, Darmstadt, Germany). Total phospholipid concentration was estimated by phosphorus assay [44]. To obtain DOX-LPs and DOX-SF-LPs, DOX loading was performed using the transmembrane pH gradient method [23]. Briefly, the lipid film was hydrated with 250 mM ammonium sulfate, pH 7.0 and concentrated by three centrifugations at 1000× *g* for 30 min using a centrifugal filter unit. The obtained liposome sample was incubated with 1 mg/mL DOX in 0.9% sodium chloride for 1 h at 60 °C. Then, to separate nonencapsulated drug, the mixed solution was subjected to three centrifugations using ultra centrifugal filters for 30 min. Finally, the liposome samples were resuspended in PBS, pH 7.4 and stored at 4 °C until used.

### 3.5. Characterization of Uncoated Liposomes and SF-LPs

#### 3.5.1. Particle Size and Zeta Potential of Liposomes and SF-LPs

The liposomal samples were diluted 10-fold in 1 mL of PBS, pH 7.4 prior to the analysis. The particle sizes and zeta potentials of uncoated liposomes and SF-LPs were measured using a Zetasizer Nano ZS (Malvern Instruments Ltd., Malvern, UK) at 25 °C. The measurement was performed in triplicate.

#### 3.5.2. Morphology

Liposomal samples were placed on copper grids and stained with 1% phosphotungstic acid. The morphology of samples was visualized under a transmission electron microscope (TEM, JEOL JEM-2010, Tokyo, Japan) at an accelerating voltage of 200 kV.

### 3.6. Determination of Encapsulation Efficiency

To determine the amount of DOX encapsulated in DOX-LPs or DOX-SF-LPs, the nonencapsulated DOX was separated by centrifugation. The sample was then diluted 20-fold in PBS, pH 7.4 with the addition of 10% Triton X-100 to disrupt the liposomal membranes. DOX concentrations in both encapsulated and nonencapsulated DOX were measured by determination of absorbance at 480 nm. The encapsulation efficiency was calculated as follows:

EE (%) = [W_encap_/(W_encap_ + W_free_)] × 100 (1)

where W_encap_ is the weight (mg) of DOX encapsulated inside the liposome, and W_free_ is the weight (mg) of nonencapsulated DOX.

### 3.7. In Vitro Drug Release

The in vitro release of DOX was investigated in PBS, pH 7.4 at 37 °C. In each experiment, DOX-LPs or DOX-SF-LPs (0.5 mL) were transferred into SnakeSkin dialysis tubing (10 kDa MWCO, Thermo Fischer Scientific, Waltham, MA, USA), which was sealed and immersed in 49.5 mL of PBS, pH 7.4 at 37 °C. At the predetermined time, 1 mL of the sample was withdrawn and replaced with an equal volume of fresh PBS, pH 7.4. The samples were collected at each time point for a total period of 21 days. All fractions of released DOX were quantified by measurement of absorbance at 480 nm. 

The obtained data were fitted into the mathematical models to define the release profile of DOX using GraphPad Prism software (version 6, La Jolla, CA, USA). Mathematical models were applied to the data fitting to define the drug release profile. The Korsmeyer–Peppas model is considered the most well-known model of drug release from polymeric systems [45], represented as the following equation:

M_t_/M_∞_ = K_kp_ × t^n^ (2)

where M_∞_ is the amount of drug at the equilibrium state, M_t_ is the amount of drug released over time t, K_kp_ is the Korsmeyer release rate constant which is dependent on structural and geometrical characteristics of the nanocarrier, and n is the diffusional exponent or drug release exponent, which is an important indicator for this kinetic model that determines the release mechanism of the drug and different phenomena of a matrix system.

### 3.8. Cell Viability Assay

In vitro cytotoxicity was determined using the MTT assay against a normal cell line (L929 mouse fibroblasts) and human breast cancer cell lines (MDA-MB-231 and MCF-7). These cell lines were cultured in Dulbecco’s modified Eagle medium (DMEM) supplemented with 10% fetal bovine serum (FBS), 100 U/mL penicillin, and 100 µg/mL streptomycin. Cells were seeded in a 96-well plate at a density of 1 × 10^4^ cells per well at 37 °C, 5% CO_2_. After 24 h incubation, 100 µL of cell growth medium was discarded and replaced with 100 µL of fresh culture medium containing the sample. The MTT assay was performed after 24 h and 48 h incubation. Then, the culture medium was replaced with 10 µL of MTT solution (0.5 mg/mL). The plates were incubated for 3 h at 37 °C, 5% CO_2_ followed by the removal of the supernatants. DMSO (100 µL) was added to dissolve the formazan crystals. Absorbance was measured at 540 nm using a microplate reader (SpectraMax i3X, Molecular Devices, San Jose, CA, USA). Cells incubated with culture medium or PBS were used as a negative control. The percentage of cell viability was calculated as follows:

% Cell viability = [A_540_ (sample)/A_540_ (negative control)] × 100 (3)

where A_540_ (sample) is the absorbance of each sample at 540 nm and A_540_ (negative control) is the absorbance of the negative control at 540 nm.

## 4. Conclusions

This work demonstrated SF-LPs as a biomimetic drug delivery system with the possibility of modifying drug release kinetics while reducing the potential cytotoxicity of the chemotherapeutic drug and cationic lipid on normal cells. Liposomes were modified by positive charges of SA to provide electrostatic interactions with the addition of methanol to achieve an effective SF coating. SF-LPs loaded with DOX using 1% SF (*w*/*v*) were prepared using the transmembrane pH gradient method to obtain a high %EE of DOX, which provided appropriate physicochemical characteristics as a drug nanocarrier and displayed long-term drug release behavior. Furthermore, DOX-SF-LPs exhibited anticancer activity against MCF-7 and MDA-MBA231 cells after 48 h and 72 h of treatment. The physicochemical properties of SF-LPs still need to be further optimized to improve drug efficacy in cancer therapy.

## Figures and Tables

**Table 1:** Physicochemical properties of liposomes and encapsulation efficiency (%EE) of DOX.

SF Concentration (%)	Size (nm)	PDI	Zeta Potential (mV)	%EE of DOX
0	136.49 ± 6.99	0.121 ± 0.020	16.4 ± 2.2	95.92 ± 2.47
0.5	145.92 ± 10.73 *	0.135 ± 0.028 ^n.s.^	14.0 ± 2.1 *	94.87 ± 2.92 ^n.s.^
1	151.00 ± 12.77 **	0.151 ± 0.045 ^n.s.^	15.0 ± 2.2 ^n.s.^	91.54 ± 5.14 ^n.s.^
2	191.63 ± 61.09 *	0.238 ± 0.159 *	9.7 ± 6.5 **	88.29 ± 9.17 ^n.s.^

Note: ** *p* < 0.01; * *p* < 0.05; n.s. = not significant, compared with uncoated LPs, SF = 0%.

**Table 2:** Fitting parameters of drug release in SF-LPs using Korsmeyer–Peppas kinetic model.

Samples	Long-Term Release (21 Days)		
	K_kp_	*n*	*R* ^2^
Uncoated DOX-LPs	12.80	0.2877	0.9913
DOX-SF-LPs, 0.5% SF	6.953	0.3287	0.9930
DOX-SF-LPs, 1.0% SF	7.125	0.3192	0.9939
DOX-SF-LPs, 2.0% SF	7.993	0.3350	0.9943

K*_kp_* is the rate constant incorporating the structural and geometric characteristics of the device under investigation; *n* is the drug release exponent that determines the release mechanism of the drug.

## Data Availability

The data presented in this study are available on request from the corresponding author.

## Supplementary Materials

The following are available online, Figure S1: Representative TEM images of SF-LPs (1% SF). (**A**) SF-LPs without stearylamine (SA) (**B**) SF-LPs containing SA on the surface before coating using SF at 1%. In the presence of SA, SF-LPs showed the relatively smooth surface compared to SF-LPs without SA, Table S1: Physicochemical properties of SF-LPs with and without methanol addition during SF coating.

## Sample Availability

Samples of the compounds are available from the authors.
