# Supplementary material for: Silk Fibroin-Coated Liposomes as Biomimetic Nanocarrier for Long-Term Release Delivery System in Cancer Therapy
Source: Molecules. 2021 Aug 15;26(16):4936. doi: 10.3390/molecules26164936 (PMC8398433; doi:10.3390/molecules26164936)
Supplement: Supplementary file 1 [file molecules-26-04936-s001.zip › molecules-1318170-supplementary.pdf]

Supplementary Material

Silk Fibroin Coated Liposomes as Biomimetic Nanocarrier for Long-term Release Delivery System in Cancer Therapy

Chanon Suyamud [1, 3], Chanita Phetdee [2, 3], Thanapak Jaimalai [3] and Panchika Prangkio [3, 4*]

1 Master's Degree Program in Chemistry, Faculty of Science, Chiang Mai University, Chiang Mai 50200, Thailand; chanon_suy@cmu.ac.th
2 Doctor of Philosophy Program in Chemistry, Faculty of Science, Chiang Mai University, Chiang Mai 50200, Thailand; chanita_phet@cmu.ac.th
3 Department of Chemistry, Faculty of Science, Chiang Mai University, Chiang Mai 50200, Thailand; thanapak.nj@gmail.com (T.J.); panchika.p@cmu.ac.th (P.P.)
4 Center of Excellence in Materials Science and Technology, Faculty of Science, Chiang Mai University, Chiang Mai 50200, Thailand
* Correspondence: panchika.p@cmu.ac.th; Tel.: +66-053-943-341

Table S1. Physicochemical properties of SF-LPs with and without methanol addition during SF coating

Sample	Size (nm)	PDI
Uncoated LPS	136.24 ± 0.52	0.108 ± 0.000
SF-LPS, 0.5% SF	150.97 ± 0.57	0.214 ± 0.001
SF-LPS, 0.5% SF without MeOH	258.40 ± 5.60	0.216 ± 0.014
SF-LPS, 1.0% SF	136.24 ± 2.30	0.220 ± 0.004
SF-LPS, 1.0% SF without MeOH	225.21 ± 27.9	0.496 ± 0.096
SF-LPS, 2.0% SF	148.55 ± 2.13	0.222 ± 0.016
SF-LPS, 2.0% SF without MeOH	343.25 ± 45.5	0.738 ± 0.046

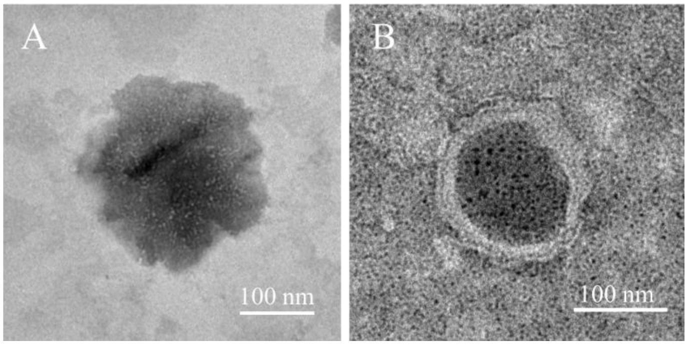

Figure S.1 Representative TEM images of SF-LPs (1% SF). **(A)** SF-LPs without stearylamine (SA) **(B)** SF-LPs containing SA on the surface before coating using SF at 1%. In the presence of SA, SF-LPs showed the relatively smooth surface compared to SF-LPs without SA.
